# Threshold Dynamics in a Model for Zika Virus Disease with Seasonality

**DOI:** 10.1007/s11538-020-00844-6

**Published:** 2021-02-17

**Authors:** Mahmoud A. Ibrahim, Attila Dénes

**Affiliations:** 1 Bolyai Institute, University of Szeged, Aradi vértanúk tere 1., Szeged, 6720 Hungary; 2 Department of Mathematics, Faculty of Science, Mansoura University, Mansoura, 35516 Egypt

**Keywords:** Periodic epidemic model, Zika virus (ZIKV), Global stability, Uniform persistence

## Abstract

We present a compartmental population model for the spread of Zika virus disease including sexual and vectorial transmission as well as asymptomatic carriers. We apply a non-autonomous model with time-dependent mosquito birth, death and biting rates to integrate the impact of the periodicity of weather on the spread of Zika. We define the basic reproduction number ${\mathscr {R}}_{0}$ as the spectral radius of a linear integral operator and show that the global dynamics is determined by this threshold parameter: If ${\mathscr {R}}_0 < 1,$ then the disease-free periodic solution is globally asymptotically stable, while if ${\mathscr {R}}_0 > 1,$ then the disease persists. We show numerical examples to study what kind of parameter changes might lead to a periodic recurrence of Zika.

## Introduction

Zika virus disease or Zika fever is a mosquito-borne disease caused by the Zika virus (ZIKV). This *Flavivirus* was first identified in monkeys in Uganda in 1947 (Dick et al. 1952), then identified in humans in 1952 in Uganda and Tanzania (Smithburne 1952). The first cases of Zika infection in South America were detected in Brazil in spring 2015 and several further countries from the region reported Zika cases in the same year. Zika virus is chiefly spread in tropical and subtropical regions by the bite of infected female mosquitoes from the *Aedes* genus (by *Aedes aegypti* above all) (see, e.g., Petersen et al. 2016), the same species that is responsible for dengue, chikungunya and yellow fever transmission. Zika virus is also spread via sexual contacts, principally from men to women (Magalhaes et al. 2018). Studies suggest that ZIKV might remain in male genital secretions for a longer period (up to 6 months) than in other bodily fluids, hence, in this way, a transmission of the disease is possible even several months after recovery (Mead et al. 2018). Mothers can transmit the disease to their fetus during pregnancy or during delivery. This transmission might result in microcephaly (a medical condition with improper brain development and head size smaller than normal) and further congenital malformations. These are collectively denominated as congenital Zika syndrome. The incubation period of Zika virus disease is around 3–14 days. Most of the infected people do not show any symptoms or only mild ones including fever, rash, muscle and joint pain, conjunctivitis and headache, in general lasting for 2–7 days (WHO 2015).

A number of sophisticated mathematical models for the spread of Zika virus disease have been previously developed, see e.g. Brauer et al. (2016); Padmanabhan et al. (2017); Baca-Carrasco and Velasco-Hernández (2016). Gao et al. (2016) presented an autonomous compartmental model of Zika spread considering mosquito-borne and sexual transmission proposing an $SEIR$-type model for the human population with *S*, *E* and *I* compartments for vectors. They separated asymptomatically infected humans from those who had symptoms. Sasmal et al. (2018) established a stage-structured model to study the effect of sexual transmission. Caminade et al. (2016) and Mordecai et al. (2017) formulated a compartmental model of Zika transmission which considers the importance of weather and climate changes. In Dénes et al. (2019) a non-autonomous model was established considering most of the important features regarding Zika transmission: sexual and vector-borne transmission, the role of asymptomatically infected humans, the prolonged period of infectiousness after recovery and assessed the importance of the seasonality of weather. In (Ibrahim and Dénes 2019), this model was extended to improve the estimation of microcephaly risk due to Zika. However, most models so far have not considered seasonality, although the number of mosquitoes—and thus the number of infections—is highly dependent on the periodically changing weather circumstances. Hence, in the present work, we establish and study a model with nine compartments describing the spread of Zika virus disease in a periodically changing environment: we set the mosquito birth and death rates as well as the biting rates to be periodic with 1 year as period, following the annual change of weather. The study of such models was initiated and further extended in (Bacaër and Guernaoui 2006; Wang and Zhao 2008; Rebelo et al. 2011; Bacaër and Ait Dads 2011), where a general definition was introduced for the basic reproduction number of periodic compartmental models, defined as the spectral radius of an integral operator acting on the space of continuous periodic functions and the reproduction number was also shown to be a threshold parameter for the local stability of the disease-free periodic solution. Since then, several papers have used the methods introduced in the above works; see, e.g., (Bakary et al. 2018; Zhang and Zhao 2007; Wang et al. 2013; Liu et al. 2010; Nakata and Kuniya 2010).

Our aim is to determine the basic reproduction number for our newly established periodic model which serves as a threshold parameter regarding the persistence of the disease. In the analysis we follow the methods established in the above-cited papers, however, the techniques need to be adapted to the present model including both human–human and mosquito–human transmission. Further, it is an utmost important question to know what might lead to a regular recurrence of the epidemic. Several vector-borne diseases—malaria, dengue, chikungunya—tend to reappear from year to year, following the annual periodicity of weather. Up to now, unlike these diseases, after 1–3 major outbreaks in following years in various countries, Zika has not shown a periodic recurrence. Our hope is that our model might help to understand which changes in the parameters may contribute to such a phenomenon. This is especially important in the days of climate change, which might provoke important modifications in the mosquito-related parameters. Furthermore, other factors like mutations of the virus might also change sexual transmission rates as well.

The paper is organized as follows. In Sect. 2, we introduce our periodic compartmental model for Zika fever transmission. In Sect. 3, we determine the basic reproduction number and study the local asymptotic stability of the disease-free periodic solution. In Sect. 4, we study the global stability of the disease-free equilibrium in the case of ${\mathscr {R}}_0<1$ the persistence of the disease in case of ${\mathscr {R}}_0>1$. We also calculate the basic reproduction number of the time-constant variant of the model. In Sect. 5, we present a case study for two South American countries. We estimate the parameter values for both countries and perform sensitivity analysis to determine the parameters which have the largest effect on the outcome of the epidemic. We provide numerical simulations to study the possible effects of an alteration of various parameters to see what kind of changes might lead to an annual recurrence of the disease. The paper is closed by a discussion.

## Mathematical Model

We divide the total human population into six compartments: susceptible $S_h(t)$, exposed $E_h(t)$, symptomatically infected $I_s(t)$, asymptomatically infected $I_a(t)$, convalescent $I_r(t)$, and recovered *R*(*t*) at time $t > 0$, while the vector population is divided into three classes: susceptible $S_v(t)$, exposed $E_v(t)$, and infectious $I_v(t)$ individuals.

The total human population $$N_h(t) $$ and the total mosquito population $$N_v(t)$$ are given by:

$$\begin{aligned} \begin{aligned} N_h(t)={}&S_h(t)+E_h(t)+I_a(t)+I_s(t)+I_r(t)+R(t),\\ N_v(t)={}&S_v(t)+E_v(t)+I_v(t). \end{aligned} \end{aligned}$$

**Fig. 1:**
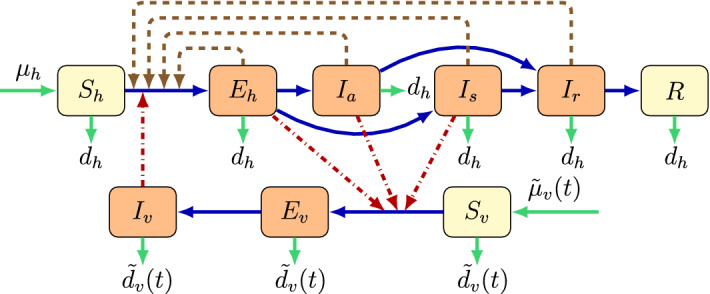
(Color figure online) Zika virus dynamics spread including vectorial and sexual transmission. Brown nodes are infectious and yellow nodes are non-infectious. Blue solid arrows show the progression of infection, while brown dashed arrows show direction of human-to-human transmission and red dash-dotted arrows show direction of transmission between humans and mosquitoes. Green arrows show birth and death

Our model takes the form

1 $$\begin{aligned} S_{h}'(t)={}&\mu _h - \beta \frac{\tau _e E_h(t) + \tau _a I_a(t)+I_s(t)+\tau _r I_r(t)}{N_h(t)} S_{h}(t)-d_h S_h(t)\nonumber \\&-\frac{{\tilde{\alpha }}_h(t)}{N_h(t)} I_v(t) S_{h}(t), \nonumber \\ E'_{h}(t)={}&\beta \frac{\tau _e E_h(t) +\tau _a I_a(t)+I_s(t)+ \tau _r I_r(t)}{N_h(t)} S_{h}(t)+ \frac{{\tilde{\alpha }}_h(t)}{N_h(t)} I_v(t)S_{h}(t)\nonumber \\&-\nu _h E_h(t)-d_h E_h(t),\nonumber \\ I_{a}'(t)={}&q \nu _h E_h(t)-\gamma _a I_a(t)-d_h I_a(t),\nonumber \\ I_{s}'(t)={}&(1-q)\nu _h E_h(t)-\gamma _s I_s(t) - d_h I_s(t),\nonumber \\ I_{r}'(t)={}&\gamma _a I_a(t)+\gamma _s I_s(t)-\gamma _r I_r(t)- d_h I_r(t),\nonumber \\ R'(t)={}&\gamma _r I_r(t)-d_h R(t),\nonumber \\ S_{v}'(t)={}&{\tilde{\mu }}_v(t) - {\tilde{\alpha }}_v(t) \dfrac{\eta _e E_h(t) +\eta _a I_a(t)+I_s(t)}{N_h(t)} S_{v}(t) -{\tilde{d}}_v(t) S_v(t),\nonumber \\ E_{v}'(t)={}&{\tilde{\alpha }}_v(t) \dfrac{\eta _e E_h(t) +\eta _a I_a(t)+ I_s(t)}{N_h(t)} S_{v}(t) -\nu _v E_v(t) - {\tilde{d}}_v(t) E_v(t),\nonumber \\ I_{v}'(t)={}&\nu _v E_v(t)- {\tilde{d}}_v(t) I_v(t), \end{aligned}$$

where ${\tilde{\mu }}_v(t)$, ${\tilde{\alpha }}_h(t)$, ${\tilde{\alpha }}_v(t)$ and ${\tilde{d}}_v(t)$ denote mosquito birth rate, transmission rate from an infectious mosquito to a susceptible human, the transmission rate from infected humans to susceptible mosquitoes and mosquito death rate, respectively. In our model, we assumed ${\tilde{\mu }}_v(t)$, ${\tilde{\alpha }}_h(t)$, ${\tilde{\alpha }}_v(t)$ and ${\tilde{d}}_v(t)$ to be continuous, positive $\omega$-periodic functions. An individual may progress from susceptible ($S_h$) to exposed ($E_h$) upon contracting the disease. An exposed individual moves either to the symptomatically infected class $I_s$ or to the asymptomatically infected class $I_a$, depending on whether that person shows symptoms or not. Infected people with or without symptoms move to the convalescent compartment $I_r$ including those who have already recovered, but who can still transmit the disease via sexual contact. After the convalescent period, one moves to the recovered compartment *R*. Mosquitoes may progress from susceptible ($S_v$) to exposed ($E_v$) and then to infectious ($I_v$) class. The description of the model parameters is summarized in Table 1, while the transmission diagram of the model can be seen in Fig.  1. We note that although the population is non-constant, the recruitment term in our model is given as $\mu _h$ instead of $\mu _h N_h$, as the countries studied in this work can be expected to be close to constant within a reasonable time interval. Doing so, we also followed among others the works Bacaër and Guernaoui (2006), Liu et al. (2010), Qu et al. (2017), Wang et al. (2013). We emphasize that a similar model was established and studied in Dénes et al. (2019), which also included differentiation of the two sexes. However, no stability analysis was performed in that paper, only numerical results were presented.

**Table 1:** Description of parameters of model 1

Parameter	Description
$\mu _h$	Human birth rate
$d_h$	Human death rate
$\beta$	Transmission rate from infected humans to susceptible humans
$\alpha _h$	Baseline value of transmission rate from mosquitoes to humans
$\alpha _v$	Baseline value of transmission rate from humans to mosquitoes
*q*	Proportion of asymptomatic infections
$\tau _e,\tau _a,\tau _r$	Relative human-to-human transmissibility of (exposed, asymptomatic and convalescent) humans to symptomatic humans
$\eta _e,\eta _a$	Relative human-to-mosquito transmissibility of (exposed and asymptomatically infected) humans to symptomatically infected humans
$\gamma _a$	Progression rate from $I_a$ to $I_r$
$\gamma _s$	Progression rate from $I_s$ to $I_r$
$\gamma _r$	Recovery rate of convalescent humans
$\nu _h$	Human incubation rate
$\nu _v$	Mosquitoes incubation rate
$\mu _v$	Baseline value of mosquito birth rate
$d_v$	Baseline value of mosquito death rate

We define $X_h=( S_h, E_h , I_{a}, I_{s}, I_{r}, R)$ and the functions $g_1, g_2, g_3 \in C({\mathbb {R}}^6_+,{\mathbb {R}}_+)$ by

2 $$\begin{aligned} \begin{aligned}&g_1( X_h)={}{\left\{ \begin{array}{ll} 0, {} &{}\text { if } X_h = (0,0,0,0,0,0), \\ \frac{\tau _e E_h + \tau _a I_a+I_s+\tau _r I_r}{S_h+ E_h + I_{a}+I_{s}+ I_{r}+ R} S_{h},{} &{}\text { if } X_h \in {\mathbb {R}}^6_+\setminus \{(0,0,0,0,0,0)\}, \end{array}\right. }\\&g_2( X_h)={}{\left\{ \begin{array}{ll} 0 , {} &{}\text { if } X_h = (0,0,0,0,0,0), \\ \frac{1}{S_h+ E_h + I_{a}+I_{s}+ I_{r}+ R}S_{h}, {}&{} \text { if } X_h \in {\mathbb {R}}^6_+\setminus \{(0,0,0,0,0,0)\}, \end{array}\right. }\\&g_3( X_h)={}{\left\{ \begin{array}{ll} 0 , {} &{}\quad \text { if } X_h = (0,0,0,0,0,0), \\ \frac{\eta _e E_h(t) +\eta _a I_a(t)+I_s(t)}{S_h+ E_h + I_{a}+I_{s}+ I_{r}+ R}, {} &{}\quad \text { if } X_h \in {\mathbb {R}}^6_+\setminus \{(0,0,0,0,0,0)\}. \end{array}\right. } \end{aligned} \end{aligned}$$

Clearly, $$g_1( X_h)$$, $$g_2( X_h)$$ and $$g_3( X_h)$$ are continuous on $${\mathbb {R}}^6_+$$. Also, $$g_1( X_h)$$, $$g_2(X_h)$$ and $$g_3( X_h)$$ are globally Lipschitz on $${\mathbb {R}}^6_+$$. By a change of variable $$N_h = S_h + E_h + I_a+ I_s+ I_r + R $$ and from (2), system (1) is equivalent to

3 $$\begin{aligned}&S_{h}'(t)={}\mu _h - \beta g_1( S_h, E_h , I_{a}, I_{s}, I_{r}, N_h ) - {\tilde{\alpha }}_h(t)g_2( S_h, E_h , I_{a}, I_{s}, I_{r}, N_h ) I_v(t)\nonumber \\&\qquad \qquad -d_h S_h(t),\nonumber \\&E'_{h}(t)={} \beta g_1( S_h, E_h , I_{a}, I_{s}, I_{r}, N_h ) + {\tilde{\alpha }}_h(t)g_2( S_h, E_h , I_{a}, I_{s}, I_{r}, N_h ) I_v(t)\nonumber \\&\qquad \qquad -\nu _h E_h(t)-d_h E_h(t),\nonumber \\&I_{a}'(t)={} q \nu _h E_h(t)-\gamma _a I_a(t)-d_h I_a(t),\nonumber \\&I_{s}'(t)={} (1-q)\nu _h E_h(t)-\gamma _s I_s(t) - d_h I_s(t), \nonumber \\&I_{r}'(t)={} \gamma _a I_a(t)+\gamma _s I_s(t)-\gamma _r I_r(t)- d_h I_r(t),\nonumber \\&N_{h}'(t)={} \mu _h -d_h N_h(t),\nonumber \\&S_{v}'(t)={} {\tilde{\mu }}_v(t) - {\tilde{\alpha }}_v(t) g_3( S_h, E_h , I_{a}, I_{s}, I_{r}, N_h ) S_{v}(t) -{\tilde{d}}_v(t) S_v(t),\nonumber \\&E_{v}'(t)={} {\tilde{\alpha }}_v(t) g_3( S_h, E_h , I_{a}, I_{s}, I_{r}, N_h ) S_{v}(t) -\nu _v E_v(t)- {\tilde{d}}_v(t) E_v(t),\nonumber \\&I_{v}'(t)={} \nu _v E_v(t)- {\tilde{d}}_v(t) I_v(t). \end{aligned}$$

We now prove the existence of a disease-free periodic solution of (3). For the human subsystem of system (3) with initial condition

$$\begin{aligned} X^0=\left( S_h(0), E_h(0), I_{a}(0), I_{s}(0), I_{r}(0),N_{h}(0),S_v(0), E_v(0), I_v(0)\right) \in {\mathbb {R}}^{9}_{+}, \end{aligned}$$

we have the linear differential equation

4 $$\begin{aligned} \frac{\mathrm{d}N_h}{\mathrm{d}t}(t)={} \mu _h -d_h N_h(t). \end{aligned}$$

One can easily see that (4) has a single equilibrium $N_{h}^*=\frac{\mu _h}{d_h}$, which is globally asymptotically stable and $N_h(t)$ is bounded.

To determine the disease-free periodic solution of (3), we study equation

5 $$\begin{aligned} S_{v}'(t)= {\tilde{\mu }}_v(t) -{\tilde{d}}_v(t) S_v(t), \end{aligned}$$

with initial value $S_v(0) \in \mathbb {R_+}$. Equation (5) has a single positive $\omega$-periodic solution $S^{*}_v(t)$, globally attractive in $\mathbb {R_+}$ and thus, system (3) has a single disease-free periodic solution $E_0=\big (N_{h}^*,0,0,0,0,N_{h}^*,S^{*}_v(t),0,0\big ).$

To formulate our next result, we introduce the notations $h^L= \sup _{t\in [ 0,\omega )} h(t)$ and $h^M= \inf _{t\in [ 0,\omega )} h(t)$ for a continuous, positive $\omega$-periodic function *h*(*t*).

### Lemma 1

There exists an $N^{*}_v= \frac{{\tilde{\mu }}_v^L}{{\tilde{d}}^L_v}>0$ such that every forward solution in $X:=\left\{ \left( S_h, E_h, I_{a}, I_{s}, I_{r}, N_h ,S_v, E_v, I_{v}\right) \in {\mathbb {R}}^9_+ : N_h\geqslant S_h+ E_h+ I_{a}+ I_{s}+ I_{r} ,\ N_v\right. \left. \geqslant S_v+E_v+I_v \right\}$ of (3) eventually enters

$$\begin{aligned} G_{N^{*}}:= & {} {} \left\{ \left( S_h, E_h, I_{a}, I_{s}, I_{r}, N_h ,S_v, E_v, I_{v}\right) \in X: N_h\right. \\&\quad \left. \leqslant N_{h}^* ,\ S_v+E_v+I_v \leqslant N^{*}_v<\infty \right\} , \end{aligned}$$

and for each $N_v(t)\geqslant N^{*}_v$, $G_{N}$ is positively invariant for (3). Further, it holds that

$$\begin{aligned} \lim _{t\rightarrow +\infty }\left( N_v(t)-S^{*}_v(t)\right) =0, \end{aligned}$$

where $$N_v(t) = S_v(t)+E_v(t)+ I_v(t)$$.

### Proof

From (3), for the mosquito subsystem, we have

$$\begin{aligned} N_{v}'(t)= {\tilde{\mu }}_v(t)-{\tilde{d}}_v(t) N_v(t)\leqslant \mu _v^L- d_v^M N_v(t) \leqslant 0, \quad \text{ if }\ N_v(t)\geqslant N^{*}_v, \end{aligned}$$

which implies that $G_N$, $N_v(t)\geqslant N^{*}_v$, is forward invariant and eventually, every positive orbit will enter $G_{N^*}$. For the second part of the proof, let us assume that

$$\begin{aligned} y(t)=N_v(t)-S^{*}_v(t), \qquad t\geqslant 0. \end{aligned}$$

We then have that

$$\begin{aligned} y'(t) = -{\tilde{d}}_v(t)y(t), \end{aligned}$$

which implies that

$$\begin{aligned} \lim _{t\rightarrow +\infty }y(t)=0. \end{aligned}$$

Hence, the proof is complete. $\square$

## Basic Reproduction Number, Local Stability

Following the technique and the notations introduced by Wang and Zhao (2008), we show the local stability of the disease-free periodic equilibrium $E_0$ of (3) for appropriate parameter values. First, we introduce the basic reproduction number ${\mathscr {R}}_0$ for system (3). Let ${\mathscr {X}}=(E_h,I_a,I_s,I_r,E_v,I_v,S_h,N_h,S_v)^T$ with ${\mathscr {F}}(t,{\mathscr {X}}(t))$, $\mathscr {V^+}(t,{\mathscr {X}}(t))$ and $\mathscr {V^-}(t,{\mathscr {X}}(t))$ denote the input rate of newly infected individuals, the input rate of individuals by other means and the rate of transfer of individuals out of compartments, respectively.

System (3) is equivalent to

6 $$\begin{aligned} {\mathscr {X}}' (t)={\mathscr {F}}(t,{\mathscr {X}} (t))-{\mathscr {V}}(t,{\mathscr {X}}(t)), \end{aligned}$$

where ${\mathscr {V}}(t,{\mathscr {X}}(t))=\mathscr {V^-} (t,{\mathscr {X}}(t))-\mathscr {V^+}(t,{\mathscr {X}}(t))$. We know that (6) has the disease-free periodic solution ${\mathscr {X}}^{*}(t)=\left( 0,0,0,0,0,0,N_{h}^*,N_{h}^*,S^{*}_v(t)\right) .$ Then by a simple computation we obtain

7 $$\begin{aligned} F(t)=&{} \begin{bmatrix} \beta \tau _e &{} \beta \tau _a &{} \beta &{} \beta \tau _r &{}0&{} {\tilde{\alpha }}_h(t) \\ 0&{}0&{}0&{}0&{} 0&{}0\\ 0&{}0&{}0&{}0&{}0 &{}0\\ 0&{}0&{}0&{}0&{}0&{}0 \\ \frac{\eta _e {\tilde{\alpha }}_v(t)}{N_{h}^*} S^{*}_{v}(t)&{}\frac{\eta _a {\tilde{\alpha }}_v(t)}{N_{h}^*} S^{*}_{v}(t)&{}\frac{{\tilde{\alpha }}_v(t)}{N_{h}^*} S^{*}_{v}(t)&{}0&{}0&{}0 \\ 0&{}0&{}0&{}0&{}0&{}0 \\ \end{bmatrix},\nonumber \\ V(t)={}&\begin{bmatrix} \nu _h + d_h&{}0&{}0&{}0&{}0&{}0 \\ -q \nu _h&{}\gamma _a + d_h&{}0&{}0&{}0&{}0 \\ -(1-q) \nu _h&{}0 &{}\gamma _s + d_h&{}0&{}0&{}0\\ 0 &{} - \gamma _a &{} -\gamma _s&{} \gamma _r + d_h &{}0&{}0 \\ 0&{}0&{}0&{}0 &{}\nu _v + {\tilde{d}}_v(t)&{}0 \\ 0&{}0&{}0&{}0&{}-\nu _v&{}{\tilde{d}}_v(t) \\ \end{bmatrix},\nonumber \\ M(t)={}&\begin{bmatrix} -d_h &{} 0 &{} 0\\ 0 &{} -d_h &{} 0\\ 0 &{} 0 &{} -{\tilde{d}}_v(t)\\ \end{bmatrix} . \end{aligned}$$

Furthermore, *F*(*t*) is non-negative, and $$-V(t)$$ is cooperative. Also $$ F(t)-V(t)$$ is irreducible for all *t*. It is straightforward to see that the conditions (A1)–(A6) are satisfied, and $${\mathscr {X}}^{*}(t)$$ is linearly asymptotically stable in the disease-free subspace

$$\begin{aligned} {\mathscr {X}}_s=(0,0,0,0,0,0,S_h,N_h,S_v) \in {\mathbb {R}}_{+}^{9}. \end{aligned}$$

Assume $Y(t,s), t \geqslant s$ is the evolution operator of the linear $\omega$-periodic system

8 $$\begin{aligned} \frac{\mathrm{d}y}{{\mathrm{d}t}} =-V (t)y. \end{aligned}$$

That is, for each $s \in {\mathbb {R}}$, the $6 \times 6$ matrix *Y*(*t*, *s*) satisfies

$$\begin{aligned} \frac{\mathrm{d}}{{\mathrm{d}t}}Y(t,s) =-V(t)Y(t,s), \qquad \forall t \geqslant s, \ Y(s,s)= I, \end{aligned}$$

where *I* is the $6 \times 6$ identity matrix. Thus, the monodromy matrix $\varPhi _{-V}(t)$ of (8) is equal to $Y(t, 0),\ t \geqslant 0$. Therefore, the condition (A7) holds.

Suppose $\phi (s)$, $\omega$-periodic in *s*, is the initial distribution of infectious individuals. Then $F(s) \phi (s)$ gives us the rate of new infections produced by the infected individuals introduced at time *s*. Given $t \geqslant s$, then $Y(t, s) F(s) \phi (s)$ supplies the distribution of those newly infected at time *s* who remain in the infected compartments at time *t*. Then

$$\begin{aligned} \psi (t):=\int _{-\infty }^{t} Y(t, s)F(s) \phi (s){\mathrm {d}s}=\int _{0}^{\infty }Y(t, t-a)F(t-a) \phi (t-a) da, \end{aligned}$$

is the distribution of accumulative new infections at time *t* due to all infected individuals $\phi (s)$ introduced at time less than *t*.

We denote by $C_\omega$ the ordered Banach space of $\omega$-periodic functions from ${\mathbb {R}}$ to ${\mathbb {R}}^{6}$, equipped with the maximum norm $\Vert \cdot \Vert _{\infty }$ and introduce the positive cone

$$\begin{aligned} C^{+}_\omega :=\{\phi \in C_\omega : \phi (t) \geqslant 0,\ \forall t \in {\mathbb {R}}\}. \end{aligned}$$

Following Wang and Zhao (2008), we then define the linear operator $L :C_\omega \rightarrow C_\omega$ as

9 $$\begin{aligned} (L \phi )(t)=\int _{0}^{\infty }Y(t, t-a)F(t-a) \phi (t-a) da, \qquad \forall t \in {\mathbb {R}}, \ \phi \in C_\omega , \end{aligned}$$

called the next infection operator. The basic reproduction number of (1) is defined as${\mathscr {R}}_0:= \rho (L)$, i.e. the spectral radius of the next infection operator *L*.

For the periodic case, let $W(t,\lambda )$ be the monodromy matrix of

$$\begin{aligned} \frac{\mathrm {d} \omega }{{\mathrm {d}t}} =\left( -V(t)+\frac{1}{\lambda } F(t)\right) \omega , \qquad \ t \in {\mathbb {R}}, \end{aligned}$$

with parameter $\lambda \in (0,\infty )$. Since *F*(*t*) is non-negative and $-V (t)$ is cooperative, we obtain that $\rho (W(\omega ,\lambda ))$ is continuous and non-increasing in $\lambda \in (0,\infty )$ and $\lim _{\lambda \rightarrow \infty } \rho (W(\omega ,\lambda ))<1$.

From the above discussion, we obtain the following result for the local asymptotic stability of the disease free periodic solution $E_0$ of our model (1).

### Theorem 1

(Wang and Zhao (2008, Theorem 2.1)) The following statements hold.

(i) If $\rho (W(\omega ,\lambda ))= 1$ has a positive solution $\lambda _0$, then $\lambda _0$ is an eigenvalue of operator *L*, and hence ${\mathscr {R}}_0> 0$.
(ii) If ${\mathscr {R}}_0> 0$, then $\lambda ={\mathscr {R}}_0$ is the unique solution of $\rho (W(\omega ,\lambda ))= 1.$
(iii) ${\mathscr {R}}_0=0$ if and only if $\rho (W(\omega ,\lambda ))< 1$ for all $\lambda >0$.

### Theorem 2

(Wang and Zhao (Wang and Zhao (2008), Theorem 2.2)) The following statements hold.

(i) ${\mathscr {R}}_0= 1$ if and only if $\rho (\varPhi _{F-V}(\omega ))=1$;
(ii) ${\mathscr {R}}_0> 1$ if and only if $\rho (\varPhi _{F-V}(\omega ))>1$;
(iii) ${\mathscr {R}}_0< 1$ if and only if $\rho (\varPhi _{F-V}(\omega ))<1$.

Hence, the disease-free periodic solution $E_0$ is locally asymptotically stable if ${\mathscr {R}}_0< 1$, and unstable if ${\mathscr {R}}_0> 1$.

### Derivation of the Basic Reproduction Number of the Autonomous Model

To calculate the basic reproduction ratio ${\mathscr {R}}^{A}_0$ of the autonomous model obtained from (3) by setting the time-dependent parameters (mosquito birth (${\tilde{\mu }}_v(t)\equiv \mu _v$) and death rates (${\tilde{d}}_v(t)\equiv d_v$) and biting rates (${\tilde{\alpha }}_h(t)\equiv \alpha _h$ and ${\tilde{\alpha }}_v(t)\equiv \alpha _v$) to constant, we follow the general approach established by Diekmann et al. (2009).

Substituting the values in the disease-free equilibrium $S^{*}_v=\frac{\mu _v}{d_v}$ in equation (7), for all $t\geqslant 0$, we obtain the Jacobian *F* given by

$$\begin{aligned} F={} \begin{bmatrix} \beta \tau _e &{} \beta \tau _a &{} \beta &{} \beta \tau _r &{}0&{} \alpha _h \\ 0&{}0&{}0&{}0&{} 0&{}0\\ 0&{}0&{}0&{}0&{}0 &{}0\\ 0&{}0&{}0&{}0&{}0&{}0 \\ \frac{\eta _e \alpha _v \mu _v d_h}{\mu _h d_v} &{}\frac{\eta _a \alpha _v \mu _v d_h}{\mu _h d_v} &{}\frac{ \alpha _v \mu _v d_h}{\mu _h d_v}&{}0&{}0&{}0\\ 0&{}0&{}0&{}0&{}0&{}0 \\ \end{bmatrix}, \end{aligned}$$

and the Jacobian *V* given by

$$\begin{aligned} V={} \begin{bmatrix} \nu _h + d_h&{}0&{}0&{}0&{}0&{}0 \\ -q \nu _h&{}\gamma _a + d_h&{}0&{}0&{}0&{}0 \\ -(1-q) \nu _h&{}0 &{}\gamma _s + d_h&{}0&{}0&{}0\\ 0 &{} - \gamma _a &{} -\gamma _s&{} \gamma _r + d_h &{}0&{}0 \\ 0&{}0&{}0&{}0 &{}\nu _v + d_v&{}0 \\ 0&{}0&{}0&{}0&{}-\nu _v&{}d_v \\ \end{bmatrix}, \end{aligned}$$

therefore the characteristic polynomial of the next generation matrix $F V^{-1}$ is

10 $$\begin{aligned} \lambda ^4 \left( \lambda ^2 - {\mathscr {R}}_{hh} \lambda - {\mathscr {R}}_{hv} {\mathscr {R}}_{vh}\right) =0, \end{aligned}$$

where

$$\begin{aligned} \begin{aligned} {\mathscr {R}}_{hh}={}&\frac{\beta }{d_h+\nu _h}\left( \tau _e +\frac{q \tau _a \nu _h }{\gamma _a+d_h } +\frac{(1-q) \nu _h }{\gamma _s+d_h} +\frac{\ \tau _r \nu _h (\gamma _s (\gamma _a+d_h)+q(\gamma _a -\gamma _s) d_h ) }{(\gamma _a+d_h) (\gamma _r+d_h) (\gamma _s+d_h) } \right) ,\\ {\mathscr {R}}_{hv}={}&\frac{\alpha _v d_h \mu _v}{d_v \mu _h (d_h+\nu _h)} \left( \eta _e +\frac{q \eta _a \nu _h }{ \gamma _a+d_h }+\frac{(1-q) \nu _h }{ \gamma _s+d_h} \right) ,\\ {\mathscr {R}}_{vh}={}&\frac{\alpha _h \nu _v}{d_v (d_v+\nu _v)}. \end{aligned} \end{aligned}$$

The characteristic polynomial therefore is the quadratic equation

11 $$\begin{aligned} \lambda ^2- {\mathscr {R}}_{hh} \lambda -{\mathscr {R}}_{hv} {\mathscr {R}}_{vh} =0. \end{aligned}$$

According to Diekmann et al. (2009), the basic reproduction number is the spectral radius of $F V^{-1}$. Thus, the basic reproduction number corresponds to the dominant eigenvalue given by the root of the quadratic equation (11)

12 $$\begin{aligned} {\mathscr {R}}^A_0={}\frac{{\mathscr {R}}_{hh}+\sqrt{{\mathscr {R}}^2_{hh}+4 {\mathscr {R}}_{hv} R_{vh}}}{2}, \end{aligned}$$

where ${\mathscr {R}}_{hh}$ and ${\mathscr {R}}_{v}= {\mathscr {R}}_{hv}{\mathscr {R}}_{vh}$ are the basic reproduction numbers corresponding to sexual transmission and vector-borne transmission, respectively. From (12), we found that ${\mathscr {R}}_{hh} +{\mathscr {R}}_{v}<1$ is the necessary and sufficient condition for ${\mathscr {R}}^A_0<1$.

## Threshold Dynamics

Here we study the global stability of the disease-free equilibrium of model (3) and the persistence of the infectious compartments. We use the general theory for the extinction or persistence of infectious given by Rebelo et al. (2011) to show that if the basic reproduction ratio ${\mathscr {R}}_0$ is less than 1, then the unique disease-free equilibrium ${\mathscr {X}}^*(t) = \big ( 0,0,0,0,0,0,N_{h}^*,N_{h}^*,S_{v}^{*}(t)\big )$ is globally asymptotically stable (G.A.S.) and the disease dies out, while if the basic reproduction ratio ${\mathscr {R}}_0$ is larger than 1, the disease persists. Moreover, we follow Zhang and Zhao (2007), Liu et al. (2010), Nakata and Kuniya (2010) and Qu et al. (2017) to prove the existence of a positive periodic solution of (3) if ${\mathscr {R}}_0>1$.

### Global Stability of the Disease-Free Equilibrium

In this subsection, we use the general method given by Rebelo et al. (2011) to show that the disease-free equilibrium is G.A.S. if ${\mathscr {R}}_0< 1$.

#### Theorem 3

If ${\mathscr {R}}_0< 1$, then the disease-free periodic solution ${\mathscr {X}}^*(t)$ of system (6) is globally asymptotically stable and if ${\mathscr {R}}_0> 1$, then it is unstable.

#### Proof

By Theorem 2, we know that ${\mathscr {X}}^*(t)$ is unstable if ${\mathscr {R}}_0> 1$ and if ${\mathscr {R}}_0< 1$, then ${\mathscr {X}}^*(t)$ is locally asymptotically stable. According to the above discussion in Sect. 3, the conditions (A1) to (A7) in Rebelo et al. (2011) are satisfied.

Moreover ${\mathscr {X}}^*(t) = \big ( 0,0,0,0,0,0,N_{h}^*,N_{h}^*,S_{v}^{*}(t)\big )$ is the unique periodic solution in the set of the disease-free states ${\mathscr {X}}_s$.

Clearly, $S(t) \leqslant N_h(t)$, for all $t \geqslant 0$. From Lemma 1, for any $\epsilon >0$, there exists $t(\epsilon )>0$ such that $S_{v}(t)\leqslant N_{v}(t)\leqslant S^*_{v}(t)+\epsilon$ for all $t \geqslant t(\epsilon )$.

Substituting into system (3), we obtain

$$\begin{aligned}&E'_{h}(t)\leqslant {}\beta \left( \tau _e E_h(t) +\tau _a I_a(t)+I_s(t)+ \tau _r I_r(t)\right) + {\tilde{\alpha }}_h(t) I_v(t) -(\nu _h +d_h) E_h(t),\\&I_{a}'(t)\leqslant {}q \nu _h E_h(t)-\gamma _a I_a(t)-d_h I_a(t),\\&I_{s}'(t)\leqslant {}(1-q)\nu _h E_h(t)-\gamma _s I_s(t) - d_h I_s(t),\\&I_{r}'(t)\leqslant {}\gamma _a I_a(t)+\gamma _s I_s(t)-\gamma _r I_r(t)- d_h I_r(t),\\&E_{v}'(t)\leqslant {}{\tilde{\alpha }}_v(t) \frac{\eta _e E_h(t) +\eta _a I_a(t)+ I_s(t)}{N^*_{h}} \big ( S^*_{v}(t)+\varepsilon \big ) -(\nu _v + {\tilde{d}}_v(t)) E_v(t),\\&I_{v}'(t)\leqslant {}\nu _v E_v(t)- {\tilde{d}}_v(t) I_v(t), \end{aligned}$$

for all $$t \geqslant t(\epsilon )$$.

Set $\mu (\epsilon ):=\min \{ S^*_{v}(\cdot )/\big (S^*_{v}(\cdot )+ \epsilon \big ) \}$. Then we have the following system:

13 $$\begin{aligned} \frac{\mathrm {d}{\tilde{U}}(t)}{\mathrm {d}t} \leqslant \left( \frac{F(t)}{\mu (\epsilon )}-V(t)\right) {\tilde{U}}(t), \qquad \forall t \geqslant t(\epsilon ), \end{aligned}$$

where ${\tilde{U}}(t)= \left( \tilde{E_h}(t),\tilde{I_a}(t),\tilde{I_s}(t), \tilde{I_r}(t),\tilde{E_v}(t),\tilde{I_v}(t)\right)$. Then ${\tilde{U}}(t) \rightarrow 0$ as $t \rightarrow \infty$ and the disease dies out.

By applying the first part of (Rebelo et al. 2011, Theorem 2), we conclude that the disease-free periodic solution ${\mathscr {X}}^*(t)$ is G.A.S. since it is G.A.S. in ${\mathscr {X}}_s$. $\square$

### Persistence of the Infective Compartments

In this subsection, we will show that the infectives are persistent if ${\mathscr {R}}_0> 1$, by using the general method given by Rebelo et al. (2011).

#### Theorem 4

If ${\mathscr {R}}_0> 1$ then system (3) is persistent with respect to $E_h$, $I_a$, $I_s$, $I_r$, $E_v$ and $I_v$.

#### Proof

Persistence of $E_h+I_a+I_s$ implies persistence of $E_h$, $I_a$ and $I_s$, and hence, persistence of $I_r$, $E_v$ and $I_v$. If there exists $\epsilon >0$ such that $\lim \inf _{t\rightarrow +\infty } \big (E_h+I_a+I_s\big )\geqslant \epsilon$, then $E_h\geqslant \frac{\epsilon }{3}-I_a-I_s$ for large *t*. Thus, from system (3), we obtain

14 $$\begin{aligned} \begin{aligned} I_{a}'\geqslant {}&q \nu _h \frac{\epsilon }{3}-(q \nu _h +\gamma _a+d_h) I_a- q \nu _h I_s,\\ I_{s}'\geqslant {}&(1-q)\nu _h \frac{\epsilon }{3}-((1-q) \nu _h +\gamma _s+d_h) I_s -(1-q) \nu _h I_a. \end{aligned} \end{aligned}$$

Thus, we have

15 $$\begin{aligned} \begin{aligned} I_{a}(t)\geqslant {}&\frac{\epsilon }{3} \frac{q \nu _h}{q \nu _h +\gamma _a+d_h} =:{} \kappa _a(\epsilon ),\\ I_{s}(t)\geqslant {}&\frac{\epsilon }{3}\frac{(1-q) \nu _h}{(1-q) \nu _h +\gamma _s+d_h}=:{} \kappa _s(\epsilon ). \end{aligned} \end{aligned}$$

By introducing the inequality (15) into the fifth equation of system (3), we get

16 $$\begin{aligned} I'_{r}\geqslant {} \gamma _a \kappa _a(\epsilon )+\gamma _s \kappa _s(\epsilon )-(\gamma _r + d_h) I_r, \end{aligned}$$

and hence,

17 $$\begin{aligned} I_{r}(t)\geqslant {} \frac{\gamma _a \kappa _a(\epsilon )+\gamma _s \kappa _s(\epsilon )}{\gamma _r + d_h} =:\kappa _r(\epsilon ). \end{aligned}$$

Consider $E_h\leqslant \epsilon$, $I_a\leqslant \epsilon$, $I_s\leqslant \epsilon$, $I_r\leqslant \epsilon$, $R\leqslant \epsilon$, $E_v\leqslant \epsilon$ and $I_v\leqslant \epsilon$ for all $t\geqslant t_0$. There exists $t_1\geqslant t_0$ such that $| N_h(t)-S^*_h|\leqslant \epsilon$ and $| N_v(t)-S^*_v(t)|\leqslant \epsilon$ for all $t\geqslant t_1$. Therefore, $S_h(t) = N_h(t)-E_h(t)-I_a(t)-I_s(t)-I_r(t)-R(t)\geqslant S^*_h-5\epsilon$ and $\ S_v(t) = N_v(t)-E_v(t)-I_v(t)\geqslant S^*_v(t)-3\epsilon$ for all $t\geqslant t_1$. From the equation for $E_v'$ of system (3), we have

18 $$\begin{aligned} \begin{aligned} E_{v}'\geqslant {}{\tilde{\alpha }}_v(t) \frac{\eta _e \frac{\epsilon }{3} +(\eta _a- \eta _e) I_a+(1-\eta _e) I_s}{N^*_h} (S^*_{v}(t)-3\epsilon ) -(\nu _v + {\tilde{d}}_v(t) )E_v.\\ \end{aligned} \end{aligned}$$

By (Rebelo et al. 2011, Lemma 1), we obtain that

19 $$\begin{aligned} \begin{aligned} E_{v}(t)\geqslant {}&\frac{{\tilde{\alpha }}^M_v \big (\eta _e \frac{\epsilon }{3} +(\eta _a- \eta _e )\kappa _a(\epsilon )+(1-\eta _e) \kappa _s(\epsilon )\big ) ({S^*}^M_{v}(t)-3\epsilon )}{2N^*_h(\nu _v + {\tilde{d}}^L_v )}=:\kappa _e(\epsilon ). \end{aligned} \end{aligned}$$

Substituting the inequality (19) into the equation for $I_v'$ of system (3), we obtain

20 $$\begin{aligned} I_{v}'\geqslant {} \nu _v \kappa _e(\epsilon )- {\tilde{d}}_v(t) I_v, \end{aligned}$$

and again by (Rebelo et al. 2011, Lemma 1), we have

21 $$\begin{aligned} I_{v}(t)\geqslant {} \frac{\nu _v \kappa _e(\epsilon )}{2 {\tilde{d}}^L_v}=:K_v(\epsilon ). \end{aligned}$$

Set $\lambda (\epsilon ):=\max \{1/\big (1-\tfrac{ 5 \epsilon }{N^*_h}\big ), \max \big (S^*_{v}(\cdot )/(S^*_{v}(\cdot )- 3 \epsilon \big )) \}.$ From the equations of system (3), for sufficiently large $t\geqslant t_1$, we obtain

$$\begin{aligned} \begin{aligned} E'_{h}(t)\geqslant {}&\beta \big ( \tau _e E_h(t) +\tau _a I_a(t)+I_s(t)+ \tau _r I_r(t)+ {\tilde{\alpha }}_h(t) I_v(t)\big ) \left( 1-\tfrac{ 5 \epsilon }{N^*_h}\right) -(\nu _h +d_h) E_h(t)\\ \geqslant {}&\beta \big ( \tau _e E_h(t) +\tau _a I_a(t)+I_s(t)+ \tau _r I_r(t)+ {\tilde{\alpha }}_h(t) I_v(t)\big ) \tfrac{1}{\lambda (\epsilon )} -(\nu _h +d_h) E_h(t),\\ I_{a}'(t)\geqslant {}&q \nu _h E_h(t)-\gamma _a I_a(t)-d_h I_a(t),\\ I_{s}'(t)\geqslant {}&(1-q)\nu _h E_h(t)-\gamma _s I_s(t) - d_h I_s(t),\\ I_{r}'(t)\geqslant {}&\gamma _a I_a(t)+\gamma _s I_s(t)-\gamma _r I_r(t)- d_h I_r(t),\\ E_{v}'(t)\geqslant {}&{\tilde{\alpha }}_v(t) \big ( \eta _e E_h(t) +\eta _a I_a(t)+ I_s(t)\big ) \left( \tfrac{S^*_{v}(t)}{N^*_h}-\tfrac{ 3 \epsilon }{N^*_h}\right) -(\nu _v + {\tilde{d}}_v(t) )E_v(t)\\ \geqslant {}&{\tilde{\alpha }}_v(t) \big ( \eta _e E_h(t) +\eta _a I_a(t)+ I_s(t)\big ) \tfrac{S^*_{v}(t)}{\lambda (\epsilon )} -(\nu _v + {\tilde{d}}_v(t) )E_v(t),\\ I_{v}'(t)\geqslant {}&\nu _v E_v(t)- {\tilde{d}}_v(t) I_v(t).\\ \end{aligned} \end{aligned}$$

From Lemma 1, it is clear that condition (A8) in Rebelo et al. (2011) is satisfied. Therefore, the assumptions of (Rebelo et al. 2011, Theorem 4) are satisfied and system (3) is persistent with respect to $E_h$, $I_a$, $I_s$, $I_r$, $E_v$, and $I_v$. $\square$

### Existence of Positive Periodic Solutions

Define

$$\begin{aligned}&X:=\left\{ (S_h,E_h, I_a,I_s,I_r,N_h,S_v,E_v,I_v)\in {\mathbb {R}}^{9}_+ \right\} ,\\&X_0:=\left\{ (S_h,E_h,I_a,I_s,I_r,N_h,S_v,E_v,I_v)\in {\mathbb {R}}_+ \times {\text{ Int }}({\mathbb {R}}^{4}_+) \times {\mathbb {R}}^{2}_+ \times {\text{ Int }}({\mathbb {R}}^{2}_+) \right\} , \end{aligned}$$

and

$$\begin{aligned} \partial X_0&:=X\setminus X_0=\left\{ (S_h,E_h,I_a,I_s,I_r,N_h,S_v,E_v,I_v): E_h I_a I_s I_r E_v I_v=0\right\} . \end{aligned}$$

Let $P:{\mathbb {R}}^9_+\rightarrow {\mathbb {R}}^9_+$ be the Poincaré map associated with (3), that is,

$$\begin{aligned} P(x^0)= u(\omega , x^0 ), \quad \text {for} \ x^0 \in {\mathbb {R}}^9_+, \end{aligned}$$

where $u(t, x^0)$ is the unique solution of (3) with $u(0, x^0) = x^0$. It is easy to see that

$$\begin{aligned} P^m(x^0)={}u(m \omega , x^0 ), \qquad \forall m\geqslant 0. \end{aligned}$$

Let *d*(*x*, *y*) denote Euclidean distance in ${\mathbb {R}}^9$. The following lemma is analogous with (Liu et al. 2010, Lemma 3.1).

#### Lemma 2

If ${\mathscr {R}}_0>1$, then there exists a $\sigma ^* > 0$ such that for any $x^0 \in X_0$, with $\Vert x^0 -E_0\Vert \leqslant \sigma ^*$ we have

$$\begin{aligned} \limsup \limits _{m \rightarrow \infty } d \left( P^m(x^0), E_0 \right) \geqslant \sigma ^* . \end{aligned}$$

#### Proof

By Theorem 2, we have that $\rho (\varPhi _{F-V}(\omega ))>1$ if ${\mathscr {R}}_0 >1.$ Then, we can choose $\eta >0$ small enough such that $\rho (\varPhi _{F-V-M_{\eta }}(\omega ))>1$ where

$$\begin{aligned} M_{\eta }(t)={} \begin{bmatrix} 0&{}0&{}0&{}0&{} 0&{}0\\ 0&{}0&{}0&{}0&{} 0&{}0\\ 0&{}0&{}0&{}0&{}0 &{}0\\ 0&{}0&{}0&{}0&{}0&{}0 \\ \frac{2\eta }{N^*_h+\eta } \eta _e {\tilde{\alpha }}_v(t) &{} \frac{2\eta }{N^*_h+\eta } \eta _a {\tilde{\alpha }}_v(t) &{} \frac{2\eta }{N^*_h+\eta } {\tilde{\alpha }}_v(t)&{} 0 &{}0&{}0 \\ 0&{}0&{}0&{}0&{}0&{}0 \\ \end{bmatrix}. \end{aligned}$$

The equation $\frac{\mathrm{d}S_h}{\mathrm{d}t}={} \mu _h -d_h S_h$ has a unique equilibrium $S^*_h={} N_{h}^*$ which is a global attractor in $\mathbb {R_+}.$

The perturbed system

22 $$\begin{aligned} \begin{aligned} \frac{\mathrm {d}\hat{S_h}(t)}{\mathrm {d}t}={}(\mu _h-\beta \sigma _1-{\tilde{\alpha }}_h(t) \sigma _2) -d_h \hat{S_h}(t),\\ \end{aligned} \end{aligned}$$

has a unique solution

$$\begin{aligned} \hat{S_h}(t,\sigma _1,\sigma _2)={} e^{-d_h t}\left( \hat{S_h}(0,\sigma _1,\sigma _2)+\int _{0}^{t}e^{-d_h s}\left( \mu _h-\beta \sigma _1-\alpha _h(s) \sigma _2\right) \,{\mathrm {d}s}\right) , \end{aligned}$$

through any initial value $\hat{S_h}(0,\sigma _1,\sigma _2)$, and it has a unique periodic solution

$$\begin{aligned} \hat{S^*_h}(t,\sigma _1,\sigma _2)={} e^{-d_h t}\left( \hat{S^*_h}(0,\sigma _1,\sigma _2)+\int _{0}^{t}e^{-d_h s}\left( \mu _h-\beta \sigma _1-\alpha _h(s) \sigma _2\right) \,{\mathrm {d}s}\right) , \end{aligned}$$

where

$$\begin{aligned} \hat{S^*_h}(0,\sigma _1,\sigma _2)={} \frac{\int _{0}^{\omega }e^{-d_h s}\left( \mu _h-\beta \sigma _1-\alpha _h(s) \sigma _2\right) \,{\mathrm{d}s}}{e^{-d_h \omega }-1}. \end{aligned}$$

It is clear that $| \hat{S_h}(t,\sigma _1,\sigma _2) - \hat{S^*_h}(t,\sigma _1,\sigma _2)| \rightarrow 0$ as $t \rightarrow \infty$, and from this we obtain that $\hat{S^*_h}(t,\sigma _1,\sigma _2)$ is globally attractive on $\mathbb {R_+}$. One can easily see that $\hat{S^*_h}(0,\sigma _1,\sigma _2)$ is continuous in $\sigma _1$ and $\sigma _2$. As the solution $\hat{S^*_h}(t,\sigma _1,\sigma _2)$ depends continuously on the initial condition and the parameter values, we obtain that $\hat{S^*_h}(t,\sigma _1,\sigma _2) > S^*_h -\eta$ holds for sufficiently small $\sigma _1$ and small $\sigma _2$, and all $t \in \left[ 0,\omega \right]$. By the periodicity of $\hat{S^*_h}(t,\sigma _1,\sigma _2)$ and constant $S^*_h -\eta$, we see that $\hat{S^*_h}(t,\sigma _1,\sigma _2) > S^*_h -\eta$ holds for sufficiently small $\sigma _1$ and small $\sigma _2$, and all $t \geqslant 0$.

Now, let us consider the following perturbed equation

23 $$\begin{aligned} \begin{aligned} \frac{\mathrm{d}\hat{S_v}(t)}{\mathrm{d}t}={}&{\tilde{\mu }}_v(t)-({\tilde{\alpha }}_v(t) \sigma _3 +{\tilde{d}}_v(t)) \hat{S_v}(t). \end{aligned} \end{aligned}$$

The Poincaré map associated with (23) has a unique positive fixed point $\hat{S^*_v}(0, \sigma _3)$ which is globally attractive in $\mathbb {R_+}$. Applying the implicit function theorem, we get that $\hat{S^*_v}(0,\sigma _3)$ is continuous in $\sigma _3$. Thus, we further fix $\sigma _3>0$ small enough such that

$$\begin{aligned} \hat{S^*_v}(t,\sigma _3)> S^*_v -\eta . \end{aligned}$$

By the continuous dependence of the solutions on the parameters and initial values and by choosing

$$\begin{aligned} \sigma :=\min \left\{ \sigma _1,\sigma _2,\sigma _3 \right\} , \end{aligned}$$

there exists a $\sigma ^*> 0$ such that for all $x^{0} \in X_0$ with $\Vert x^0 -E_0\Vert \leqslant \sigma ^*,$ it holds that

$$\begin{aligned} \Vert u(t,x^0) -u(t,E_0)\Vert \leqslant \sigma , \qquad \text{ for } \ 0\leqslant t\leqslant \omega . \end{aligned}$$

We further claim that

24 $$\begin{aligned} \limsup \limits _{m \rightarrow \infty } d \left( P^m(x^0 \right) \geqslant \sigma ^*. \end{aligned}$$

Suppose, by contradiction, that (24) does not hold. Then we have

25 $$\begin{aligned} \limsup \limits _{m \rightarrow \infty } d \left( P^m(x^0), E_0 \right) < \sigma ^* , \end{aligned}$$

for some $x^0 \in X_0$. Without loss of generality, we assume that $d \left( P^m(x^0), E_0 \right) < \sigma ^*$, for all $m \ge 0$. Then, from the above discussion, we have that

$$\begin{aligned} \Vert u(t,P^{m}(x^0)) -u(t,E_0)\Vert <\sigma , \qquad \forall m \ge 0,\ t\in \left[ 0,\omega \right] . \end{aligned}$$

For any $t\geqslant 0,$ let $t=m\omega + t_1,$ where $t_1 \in \left[ 0,\omega \right)$ and $m=[\frac{t }{\omega }]$, which is the greatest integer less than or equal to $\frac{t}{\omega }$. Then, we get

$$\begin{aligned} \Vert u(t,x^0) -u(t,E_0)\Vert =\Vert u(t_1,P^{m}(x^0)) -u(t_1,E_0)\Vert < \sigma , \quad \forall t \geqslant 0. \end{aligned}$$

Set

$$\begin{aligned} \left( S_h(t), E_h(t) , I_{a}(t), I_{s}(t), I_{r}(t),N_{h}(t),S_v(t), E_v(t) , I_v(t)\right) ={} u(t,x^0). \end{aligned}$$

It follows that $E_h(t)<\sigma$, $I_{a}(t)<\sigma$, $I_{s}(t)<\sigma$, $I_{r}(t)<\sigma$, $I_v(t)<\sigma$, for all $t\geqslant 0$ and from system (3), we have

26 $$\begin{aligned} \begin{aligned} \frac{dS_h(t)}{{\mathrm{d}t}}\geqslant {}&(\mu _h-\beta \sigma -{\tilde{\alpha }}_h(t) \sigma ) -d_h S_h(t),\\ \frac{dS_v(t)}{{\mathrm{d}t}}\geqslant {}&{\tilde{\mu }}_v(t)-({\tilde{\alpha }}_v(t) \sigma +{\tilde{d}}_v(t)) S_v(t). \end{aligned} \end{aligned}$$

As the periodic solution $\hat{S^*_h}(t,\sigma )$ of equation (22) is globally attractive on $\mathbb {R_+}$ and$\hat{S^*_h}(t,\sigma )> S^*_h-\eta$, we have

$$\begin{aligned} S_h(t)\geqslant S^*_h-\eta , \end{aligned}$$

for *t* large enough. Also, the fixed point $\hat{S^*_v}(t,\sigma )$ of the Poincaré map corresponding to (23) is globally attractive and $\hat{S^*_v}(t,\sigma )> S^*_v(t)-\eta ,$ there exists a *t* large enough such that

$$\begin{aligned} S_v(t,\sigma )> S^*_v(t)-\eta . \end{aligned}$$

From the equations of system (3), for sufficiently large *t*, we obtain

$$\begin{aligned} \begin{aligned} E'_{h}(t)\geqslant {}&\beta \left( \tau _e E_h(t) +\tau _a I_a(t)+I_s(t)+ \tau _r I_r(t)\right. \\&\left. + {\tilde{\alpha }}_h(t) I_v(t)\right) \left( 1-\tfrac{ 2 \eta }{N^*_h+ \eta }\right) -(\nu _h +d_h) E_h(t),\\ I_{a}'(t)={}&q \nu _h E_h(t)-\gamma _a I_a(t)-d_h I_a(t),\\ I_{s}'(t)={}&(1-q)\nu _h E_h(t)-\gamma _s I_s(t) - d_h I_s(t),\\ I_{r}'(t)={}&\gamma _a I_a(t)+\gamma _s I_s(t)-\gamma _r I_r(t)- d_h I_r(t),\\ E_{v}'(t)\geqslant {}&{\tilde{\alpha }}_v(t) \left( \eta _e E_h(t) +\eta _a I_a(t)+ I_s(t)\right) \left( \tfrac{S^*_{v}(t)}{N^*_h}-\tfrac{ 2 \eta }{N^*_h+ \eta }\right) -(\nu _v+{\tilde{d}}_v(t)) E_v(t),\\ I_{v}'(t)={}&\nu _v E_v(t)- {\tilde{d}}_v(t) I_v(t).\\ \end{aligned} \end{aligned}$$

Next we consider the system

27 $$\begin{aligned} \begin{aligned} \frac{\mathrm{d}{\hat{E}}_{h}(t)}{{\mathrm{d}t}}={}&\beta \left( \tau _e {\hat{E}}_{h}(t) +\tau _a {\hat{I}}_{a}(t)+{\hat{I}}_{s}(t)+ \tau _r {\hat{I}}_{r}(t) + {\tilde{\alpha }}_h(t) {\hat{I}}_{v}(t)\right) \left( 1-\tfrac{2 \eta }{N^*_h+ \eta }\right) \\&-(\nu _h +d_h) {\hat{E}}_{h}(t),\\ \frac{\mathrm{d}{\hat{I}}_{a}(t)}{{\mathrm{d}t}}={}&q \nu _h {\hat{E}}_{h}(t)-\gamma _a {\hat{I}}_{a}(t)-d_h {\hat{I}}_{a}(t),\\ \frac{\mathrm{d}{\hat{I}}_{s}(t)}{{\mathrm{d}t}}={}&(1-q)\nu _h {\hat{E}}_{h}(t)-\gamma _s {\hat{I}}_{s}(t) - d_h {\hat{I}}_{s}(t),\\ \frac{\mathrm{d}{\hat{I}}_{r}(t)}{{\mathrm{d}t}}={}&\gamma _a {\hat{I}}_{a}(t)+\gamma _s {\hat{I}}_{s}(t)-\gamma _r {\hat{I}}_{r}(t)- d_h {\hat{I}}_{r}(t),\\ \frac{\mathrm{d}{\hat{E}}_{v}(t)}{{\mathrm{d}t}}={}&{\tilde{\alpha }}_v(t) ( \eta _e {\hat{E}}_{h}(t) +\eta _a {\hat{I}}_{a}(t)+ {\hat{I}}_{s}(t)) \left( \tfrac{S^*_{v}(t)}{N^*_h}-\tfrac{ 2 \eta }{N^*_h+ \eta }\right) -(\nu _v+{\tilde{d}}_v(t)) {\hat{E}}_{v}(t),\\ \frac{\mathrm{d}{\hat{I}}_{v}(t)}{{\mathrm{d}t}}={}&\nu _v {\hat{E}}_{v}(t)- {\tilde{d}}_v(t) {\hat{I}}_{v}(t).\\ \end{aligned} \end{aligned}$$

Now we have that $\rho (\varPhi _{F-V-M_{\eta }}(\omega ))>1$. Once again by (Zhang and Zhao 2007, Lemma 2.1), there exists a positive, $\omega$-periodic function $p_2(t)$ such that $p_2(t) \exp (\xi _2 t)$ is a solution of (27) and $\xi _2 =\frac{1}{\omega } \ln \rho (\varPhi _{F-V+ M_{\eta } }(\omega ))>0$. For any $J(0) \in {\mathbb {R}}^{6}_{+}$, we can choose a real number $K^*_2 > 0$ such that $J(0) \geqslant K^*_2 p_2(0)$ where

$$\begin{aligned} J(t)=(E_h(t),I_a(t),I_s(t),I_r(t),E_v(t),I_v(t))^T. \end{aligned}$$

Applying the comparison principle (Smith and Waltman 1995, Theorem B.1), we obtain $J(t) \geqslant p_2(t) \exp (\xi _2 t)$ for all $t>0$, which implies that $\lim _{t\rightarrow \infty } E_h(t)= \infty$, $\lim _{t\rightarrow \infty } I_a(t)= \infty$, $\lim _{t\rightarrow \infty } I_s(t)=\infty$, $\lim _{t\rightarrow \infty } I_r(t)=\infty$, $\lim _{t\rightarrow \infty } E_v(t)=\infty$ and $\lim _{t\rightarrow \infty } I_v(t)= \infty$. This leads to a contradiction, which completes the proof. $\square$

#### Theorem 5

Assume that ${\mathscr {R}}_0> 1$. Then system (3) has at least one positive periodic solution, and there exists an $\varepsilon >0$ such that

$$\begin{aligned} \begin{aligned} \liminf _{t\rightarrow \infty } E_h(t)\geqslant \varepsilon ,\quad \liminf _{t\rightarrow \infty } I_a(t)\geqslant \varepsilon ,\quad \liminf _{t\rightarrow \infty } I_s(t)\geqslant \varepsilon , \\ \liminf _{t\rightarrow \infty } I_r(t)\geqslant \varepsilon ,\quad \liminf _{t\rightarrow \infty } E_v(t)\geqslant \varepsilon ,\quad \liminf _{t\rightarrow \infty } I_v(t)\geqslant \varepsilon , \\ \end{aligned} \end{aligned}$$

for all $$\left( S_h(0),E_h(0),I_a(0),I_s(0), I_r(0),N_h(0),S_v(0),E_v(0),I_v(0)\right) \in X_0$$

#### Proof

First, we prove that *P* is uniformly persistent with respect to $(X_0, \partial X_0)$, as from this, applying (Zhao 2017, Theorem 3.1.1), it follows that the solution of (3) is uniformly persistent with respect to $(X_0, \partial X_0)$.

Let $\phi =\left( S_h(0),E_h(0),I_a(0),I_s(0), I_r(0),N_h(0),S_v(0),E_v(0),I_v(0)\right) \in X_0$ be any initial condition. By solving (1) for all $t>0$, we get that

28 $$\begin{aligned} S_{h}(t)={}&e^{-\int _{0}^{t} (a_h(s)+d_h) \,{\mathrm{d}s}} \left[ S_h(0) + \textstyle \int _{0}^{t}\mu _h e^{\int _{0}^{s} (a_h(r)+d_h) \,\mathrm{d}r}\,{\mathrm{d}s}\right] >0, \end{aligned}$$

29 $$\begin{aligned} E_{h}(t)={}&e^{ - (\nu _h + d_h) t} \left[ E_h(0) + \textstyle \int _{0}^{t} a_h(s) S_h(s) e^{ (\nu _h + d_h) s}\,\right] >0, \end{aligned}$$

30 $$\begin{aligned} I_{a}(t)={}&e^{ - (\gamma _a+d_h) t} \left[ I_a(0) + q \nu _h \textstyle \int _{0}^{t} E_h(s) e^{ (\gamma _a+d_h) s}\,\mathrm{d}s\right] >0,\end{aligned}$$

31 $$\begin{aligned} I_s(t)={}&e^{ - (\gamma _s+d_h) t} \left[ I_s(0) + (1-q) \nu _h \textstyle \int _{0}^{t} E_h(s) e^{ (\gamma _s+d_h) s}\,\mathrm{d}s\right] >0,\end{aligned}$$

32 $$\begin{aligned} I_r(t)={}&e^{ - (\gamma _r+d_h) t} \left[ I_r(0) + \textstyle \int _{0}^{t} \big (\gamma _a I_a(z)+\gamma _s I_s(z) \big ) e^{ (\gamma _r+d_h) z}\,{\mathrm{d}z}\right] >0,\end{aligned}$$

33 $$\begin{aligned} S_{v}(t)={}&e^{-\int _{0}^{t} (a_v(s)+{\tilde{d}}_v(s)) \,\mathrm{d}s} \left[ S_v(0) + \textstyle \int _{0}^{t} {\tilde{\mu }}_v(s) e^{\int _{0}^{s} (a_v(r)+{\tilde{d}}_v(r)) \,\mathrm{d}r}\,\mathrm{d}s\right] >0,\end{aligned}$$

34 $$\begin{aligned} E_{v}(t)={}&e^{-\int _{0}^{t} (\nu _v+{\tilde{d}}_v(s)) \,\mathrm{d}s} \left[ E_v(0) + \textstyle \int _{0}^{t}a_v(s) S_h(s) e^{\int _{0}^{s} (\nu _v+{\tilde{d}}_v(r)) \,\mathrm{d}r} \,\mathrm{d}s\right] >0,\end{aligned}$$

35 $$\begin{aligned} I_{v}(t)={}&e^{-\int _{0}^{t} {\tilde{d}}_v(s) \,\mathrm{d}s} \left[ I_v(0) + \nu _v \textstyle \int _{0}^{t} E_v(s) e^{\int _{0}^{s} {\tilde{d}}_v(r) \,\mathrm{d}r} \,\mathrm{d}s\right] >0, \end{aligned}$$

where

$$\begin{aligned} \begin{aligned} a_h(t)= {}&\beta \frac{ \tau _e E_h(t)+ \tau _a I_a(t)+ I_s(t)+\tau _r I_r(t)}{N_h(t)} + {\tilde{\alpha }}_h (t) \frac{ I_v(t)}{N_h(t)}, \\ a_v(t)= {}&{\tilde{\alpha }}_v(t) \dfrac{ \eta _e E_h(t)+\eta _a I_a(t)+ I_s(t)}{N_h(t)}. \end{aligned} \end{aligned}$$

Hence, we get the positively invariant of $X_0$. Since *X* is also positively invariant and $\partial X_0$ is relatively closed in *X*, it gives $\partial X_0$ is positively invariant.

Furthermore, from Lemma 1, it follows that system (3) is point dissipative.

Let us introduce

$$\begin{aligned} M_{\partial } = \left\{ x^0 \in \partial X_0: P^m(x^0)\in \partial X_0, \ \forall m \geqslant 0 \right\} . \end{aligned}$$

We will apply the theory of uniform persistence developed in (Zhao 2017) (see also (Zhang and Zhao 2007, Theorem 2.3)). In order to do this, we first show that

36 $$\begin{aligned} M_{\partial } = \left\{ (S_h,0,0,0,0,N_h,S_v,0,0) : S_h \geqslant 0, \ N_h \geqslant 0 ,\ S_v \geqslant 0 \right\} . \end{aligned}$$

Let us note that $M_{\partial } \supseteq \left\{ (S_h,0,0,0,0,N_h,S_v,0,0) : S_h \geqslant 0, \ N_h \geqslant 0 ,\ S_v \geqslant 0 \right\}$. It suffices to prove that

for arbitrary initial condition $x^0 \in \partial X_0$, $E_h(n \omega ) = 0$ or $I_a(n \omega ) = 0$ or $I_s(n \omega ) = 0$ or $I_r(n \omega ) = 0$ or $E_v(n \omega ) = 0$ or $I_v(n \omega ) = 0$, for all $n \geqslant 0$.

By contradiction, assume that there exists an $n_1\geqslant 0$ for which

$$\begin{aligned} \big ( E_h(n_1 \omega ),I_a(n_1 \omega ),I_s(n_1 \omega ),I_r(n_1 \omega ),E_v(n_1 \omega ),I_v(n_1 \omega ) \big )^T >0. \end{aligned}$$

Thus, (28) implies $N_h(t)\geqslant S_h(t)>0, \ \forall t>n_1 \omega .$ Then, by replacing $t=0$ to $t = n_1 \omega$ in (28)–(35), we obtain that $S_h(t) > 0$, $E_h(t) > 0$, $I_a(t) > 0$, $I_s(t) > 0$, $I_r(t) > 0$, $N_h(t) > 0$, $S_v(t) > 0$, $E_v(t) > 0$, $I_v(t) > 0$. This is in contradiction with the positive invariance of $\partial X_0$.

By Lemma 2, *P* is weakly uniformly persistent with respect to $(X_0, \partial X_0)$. From Lemma 1, *P* has a global attractor. It follows that $E_0$ is an isolated invariant set in *X* and $W^s(E_0) \cap X_0 = \emptyset$. It is clear that every solution in $M_\partial$ converges to $E_0$ and $E_0$ is acyclic in $M_\partial$. By (Zhao 2017, Theorem 1.3.1 and Remark 1.3.1), we obtain that *P* is uniformly persistent with respect to $(X_0, \partial X_0)$. Hence, there exists an $\varepsilon >0$ such that

$$\begin{aligned} \begin{aligned}&\liminf _{t\rightarrow \infty } E_h(t)\geqslant \varepsilon ,\quad \liminf _{t\rightarrow \infty } I_a(t)\geqslant \varepsilon ,\quad \liminf _{t\rightarrow \infty } I_s(t)\geqslant \varepsilon ,\\&\liminf _{t\rightarrow \infty } I_r(t)\geqslant \varepsilon ,\quad \liminf _{t\rightarrow \infty } E_v(t)\geqslant \varepsilon ,\quad \liminf _{t\rightarrow \infty } I_v(t)\geqslant \varepsilon . \end{aligned} \end{aligned}$$

By (Zhao (2017), Theorem 1.3.6), *P* has a fixed point

$$\begin{aligned} {\bar{\phi }}= \left( {\bar{S}}_h(0), {\bar{E}}_h(0), {\bar{I}}_a(0), {\bar{I}}_s(0), {\bar{I}}_r(0), {\bar{N}}_h(0),{\bar{S}}_v(0), {\bar{E}}_v(0), {\bar{I}}_v(0)\right) \in X_0, \end{aligned}$$

and hence at least one periodic solution $u(t, {\bar{\phi }})$ of system (3) exists.

Now, we show that ${\bar{S}}_h(0)$ and ${\bar{S}}_v(0)$ are positive. If ${\bar{S}}_h(0)=0 ={\bar{S}}_v(0)$, then from (28) and (33) we get that ${\bar{S}}_h(0)> 0$ and ${\bar{S}}_v(0)> 0$ for all $t>0$. However, using the periodicity of solution, we have ${\bar{S}}_h(0)= {\bar{S}}_h(n \omega )=0$ and ${\bar{S}}_v(0)= {\bar{S}}_v(n \omega )=0$ for all $n\geqslant 1$, that leads to a contradiction. $\square$

## Case Study for Ecuador and Colombia: What Changes in the Parameters Might Lead to a Regular Recurrence of Zika Fever?

In this section, we apply our model to study the spread of Zika in Ecuador during the 2015–17 and in Colombia during the 2015–17 Zika virus epidemic. From Sect. 4, we see that ${\mathscr {R}}_0$ is a threshold parameter for the persistence of the disease in the population (see Theorems 3 and 5). The functions ${\tilde{\mu }}_v(t)$, ${\tilde{\alpha }}_h(t)$, ${\tilde{\alpha }}_v(t)$ and ${\tilde{d}}_v(t)$ are assumed to be time-periodic with one year as a period and, following e.g. Bakary et al. (2018), they are assumed to be of the form $\mu _v \cdot \big (\sin \big (\frac{2\pi }{p} t+b\big )+a\big )$, $\alpha _h \cdot \big (\sin \big (\frac{2\pi }{p} t+b\big )+a\big )$, $\alpha _v \cdot \big (\sin \big (\frac{2\pi }{p} t+b\big )+a\big )$ and $d_v \cdot \big (\cos \big (\frac{2\pi }{p} t+b\big )+a\big )$ where *p* is period length, *a*, *b* are free adjustment parameters and $\mu _v, \alpha _h,\alpha _v, d_v$ are the (constant) baseline values of the corresponding time-dependent parameters.

### Parameter Estimation for Ecuador and Colombia

To give an estimate for the values of the parameters providing the best fit, we applied Latin Hypercube Sampling, a method used in statistics to assess simultaneous variation of multiple parameters (see, e.g., McKay et al. (1979)).

**Fig. 2:**
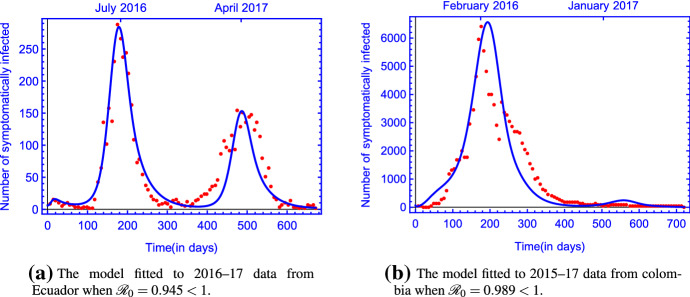
(Color figure online) The model fitted to in **a** 2016–17 data from Ecuador and in **b** 2015–17 data from Colombia when ${\mathscr {R}}_0 <1$ with parameter values in Table 2

Figure 2 shows model (1) fitted to data from Ecuador and Colombia (PAHO 2015, 2017). Our model gives a reasonably good fit for both countries, reproducing the single peak of Zika fever in Colombia and the two peaks of Zika fever experienced in Ecuador in two subsequent years. This shows that model (1) is able to reproduce the two types of outcomes of the Zika epidemic observed in South America. We note that in our simulation for Ecuador, before dying out, the epidemic shows a very minor third peak for the following year 2018. This is in accordance with real world data, as sources report a small number of Zika infections in this year. Our model slightly overestimates the number of cases in 2018, however, as in most cases, Zika fever does not cause severe symptoms and that public awareness was reduced by the decreasing number of Zika cases, probably less people visited their doctors during this third year.

Figure 2 is in accordance with the analytic results stating that the unique disease-free equilibrium $E_0$ is globally asymptotically stable when ${\mathscr {R}}_0 <1$.

**Fig. 3:**
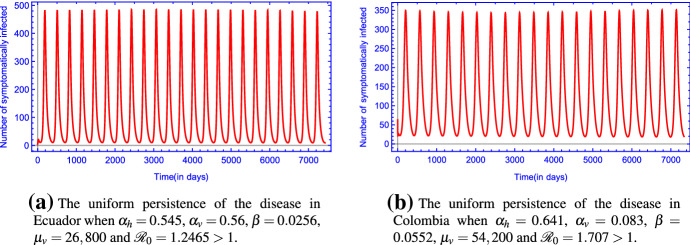
(Color figure online) The uniform persistence of the disease in **a** Ecuador and in **b** Colombia when ${\mathscr {R}}_0 >1$. The rest of the parameter values are the same as those in Table 2

By Theorem 4, system (1) is persistent with respect to the infective compartments if ${\mathscr {R}}_0 > 1$. Figure  3 shows the persistence of the disease when ${\mathscr {R}}_0>1$.

### Parameter Changes

One of our main interests was to see what changes in the parameters might lead to a regular reappearance of the epidemic. Up to now, after 1–3 consecutive years with outbreaks, Zika did not appear in high numbers again. However, in the days of climate change, one can expect that some of the parameters will change in the future as mosquitoes adapt to new circumstances and mutations of the virus appear. This might lead to a periodic annual recurrence of the epidemic, just like in the case of other mosquito-borne diseases like dengue fever or malaria. Because of the high number of parameters, it is not easy to assess rigorously which of the parameters have the most important role in the variation of the dynamics, so we only try to demonstrate the possible alterations through a couple of examples.

**Fig. 4:**
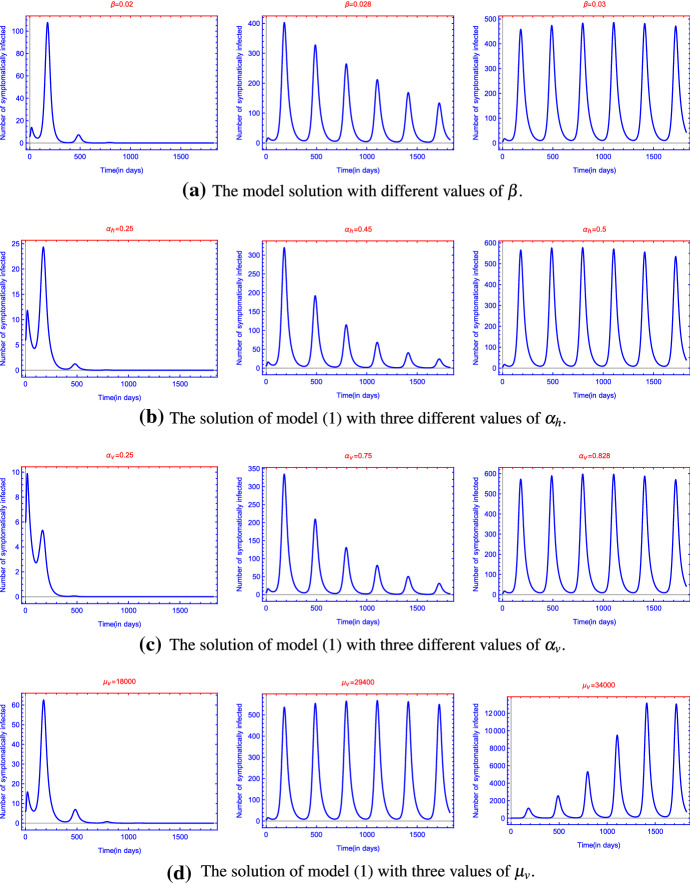
(Color figure online) The solution of model (1) with three different values of in **a** human-to-human transmission rate ($\beta$), in **b** baseline value of mosquito-to-human transmission rate ($\alpha _h$), in **c** baseline value of human-to-mosquito transmission rate ($\alpha _v$) and in **d** baseline value of mosquito birth rate ($\mu _v$). The rest of the parameter values are the same as those for Ecuador in Table 2

Our first example (see Fig. 3a) was created with the above determined parameters for the Zika epidemic in Ecuador except the mosquito-related parameters $\alpha _h,\alpha _v,\beta ,\mu _v$, i.e. we increased human-to-human and human-to-mosquito transmission rates and mosquito birth rate, while mosquito-to-human transmission rate was decreased. We can calculate numerically the value of the basic reproduction number ${\mathscr {R}}_0 = 1.2465 > 1$. In the second example (see Fig. 3b), similar changes were performed for the parameters determined for Colombia. Again, we can calculate numerically the value of the basic reproduction number ${\mathscr {R}}_0 = 1.707 > 1$. Accordingly, one can see that with these parameters, the disease compartments are persistent and the epidemic becomes endemic in the population recurring periodically every year.

In Fig. 4 we present how changes in some of the key parameters (human-to-human, human-to-mosquito and mosquito-to human transmission rates as well as mosquito birth rates) might affect the course of Zika epidemics. The simulations suggest that an increase of any of these four parameters—either due to climate change or to genetic mutation of the virus—can lead to a periodic annual reappearance of the epidemic.

**Fig. 5:**
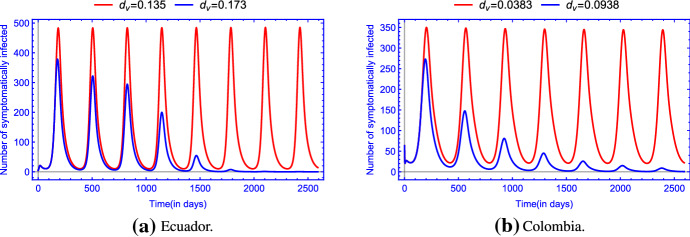
(Color figure online) Seasonal measures to control ZIKV in **a** Ecuador and in **b** Colombia. The rest of the parameter values are the same as those in Fig.  3 and Table 2

Knowing the seasonal fluctuation, one may rightly suppose that mosquito control is limited to the peak months of mosquito abundance. Hence, we also show an example where mosquito control only occurs during 5 months when the highest number of vectors are present to see whether control measures implemented only during a limited period of the year might have a sufficient effect to eradicate the disease. Mosquito killing is incorporated into the model by considering a step function multiplier of the mosquitoes’ death rate. Namely, we increase the death rate during a five-month-long period of each year. For a better assessment of the effect of additional mosquito killing, we assume a periodic recurrence of the disease, just like given in Fig.  3 and Table 2. In Fig.  5 we show some seasonal measures to control Zika virus disease both in Ecuador and Colombia. The figure suggests that even a mosquito control limited to the peak period of mosquito abundance might have a significant impact to control the disease.

### Sensitivity Analysis

Sensitivity analysis, using Partial Rank Correlation Coefficients (PRCC, see, e.g., Blower and Dowlatabadi (1994)), is carried out to determine the parameters that have the greatest influence on the dynamics of the diseases. The PRCC-based sensitivity analysis measures the effect of the parameters on the response function (in our cases, the number of infected cases), while we vary the parameters (relevant to the dynamics of the diseases in Ecuador and Colombia) in the given ranges (see Table 2).

**Fig. 6:**
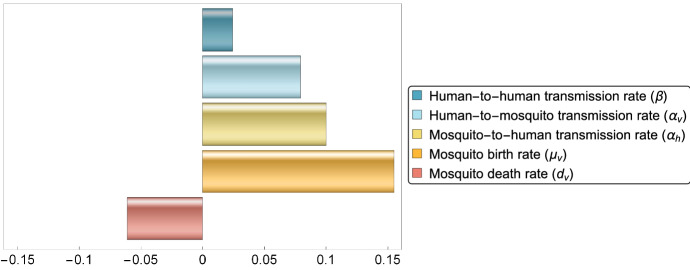
(Color figure online) Partial rank correlation coefficients of the five parameters subject to intervention measures. Parameters with positive PRCC values are positively correlated with the cumulative number of cases. Parameters with negative PRCC values are negatively correlated with the cumulative number of infections

Figure 6 shows the comparison of the PRCC values obtained for the parameters $\beta ,\alpha _h,\alpha _v,\mu _v$ and $d_v$, i.e., those parameters which can typically be affected by control measures. The results suggest that the most relevant factors in Zika transmission, and hence in the elevation of the number of infected cases are birth and death rates of mosquitoes. Spread via sexual contacts is shown to have a smaller effect; however, it is still an important factor. Based on the sensitivity analysis, we can assess that the most effective measures to reduce transmission are control of mosquito populations and protection against their bites.

**Table 2:** Parameters of model (1) and fitted values in the case of Ecuador and Colombia

Parameter	Range	Value (Ecuador)	Value (Colombia)	Source
$\mu _h$	–	608.142	1826.81	WHO (2015)
$d_h$	–	0.0000359	0.0000368	WHO (2015)
$\beta$	0.01–0.1	0.0249	0.0435	Gao et al. (2016)
$\alpha _h$	0.03–0.75	0.44	0.218	Andraud et al. (2012); Chikaki and Ishikawa (2009)
$\alpha _v$	0.09–0.75	0.727	0.347	Andraud et al. (2012); Chikaki and Ishikawa (2009)
*q*	0.75–0.9	0.765	0.824	Gao et al. (2016); Duffy et al. (2009)
$\tau _e$	0.2–1	0.796	0.382	Gao et al. (2016)
$\tau _a$	0.2–1	0.7902	0.263	Gao et al. (2016)
$\tau _r$	0.2–0.8	0.576	0.585	Gao et al. (2016)
$\eta _e$	0.2–0.7	0.636	0.653	Gao et al. (2016)
$\eta _a$	0.2–0.7	0.591	0.471	Gao et al. (2016)
$\gamma _a$	0.05–0.4	0.1169	0.165	Gao et al. (2016)
$\gamma _s$	0.2–0.5	0.1059	0.477	Bearcroft (1956)
$\gamma _r$	0.01–0.07	0.0545	0.06	Gourinat et al. (2015); Musso et al. (2015)
$\nu _h$	0.1–0.5	0.292	0.421	Bearcroft (1956)
$\nu _v$	0.08–0.125	0.106	0.0965	Andraud et al. (2012; Boorman and Porterfield 1956)
$\mu _v$	500–40, 000	25, 800	32, 200	Fitted
$1/d_v$	4–35	8	26.05	Andraud et al. (2012)
*a*	–	1.35	1.1	–
*b*	–	6.48	138.51	–

### Reproduction Numbers

**Fig. 7:**
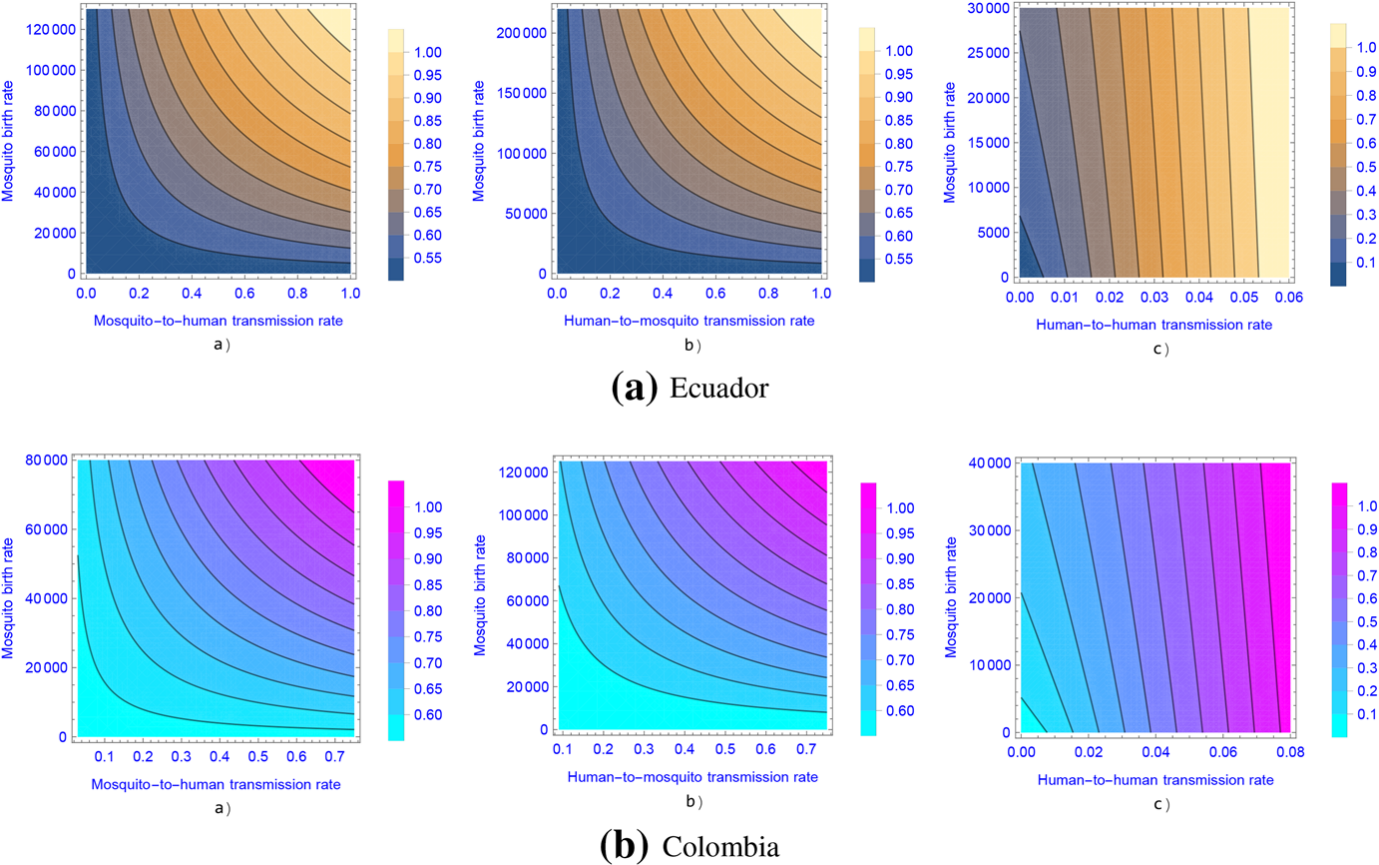
(Color figure online) Contour plot of the basic reproduction number as a function of baseline value of mosquito birth rate ($\mu _v$) and **a** baseline value of mosquito-to-human transmission rate ($\alpha _h$), **b** baseline value of human-to-mosquito transmission rate ($\alpha _v$) and **c** human-to-human transmission rate ($\beta$)

To calculate the reproduction numbers, we use the parameters as obtained in the fitting to Ecuador data in Table 2. Formula (12) provides us the basic reproduction number in any time point by substituting the parameter values. Figure 7 shows the basic reproduction number of the time-constant model w.r.t. baseline value of mosquito birth rate, baseline value of human-to-mosquito transmission rates and human-to-human transmission rate, suggesting that control of mosquito population and sexual protection both have a significant effect in Zika fever transmission. The results also imply that vector control might not be enough to contain the disease spread in case of a high sexual transmission rate.

**Fig. 8:**
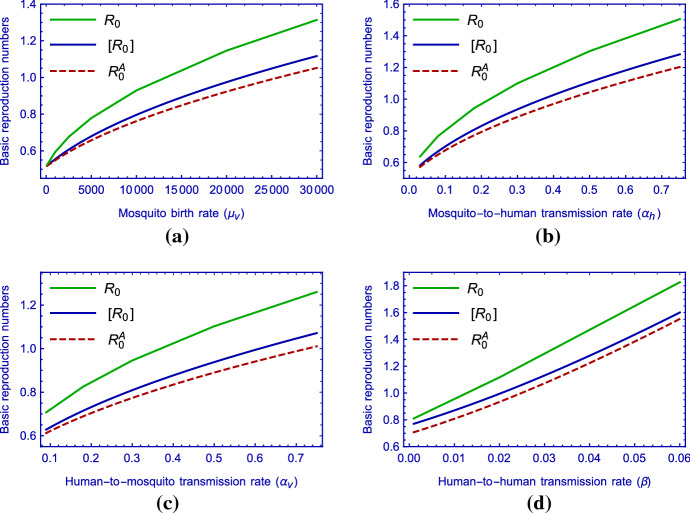
(Color figure online) The curves of the basic reproduction number ${\mathscr {R}}_0$, the time-average basic reproduction number $[{\mathscr {R}}_0]$ and the basic reproduction number of the autonomous model ${\mathscr {R}}^A_0$ versus **a** baseline value of mosquito birth rate ($\mu _v$), **b** baseline value of mosquito-to-human transmission rate ($\alpha _h$), **c** baseline value of human-to-mosquito transmission rate ($\alpha _v$) and **d** human-to-human transmission rate ($\beta$)

Further, by numerical calculations we get the curves of the basic reproduction ratio ${\mathscr {R}}_0$, the time-average basic reproduction number $[{\mathscr {R}}_0]$ (using the notation presented by Mitchell and Kribs (2017)) and the basic reproduction number ${\mathscr {R}}^{A}_0$ of the autonomous model with respect to baseline value of mosquito birth rate ($\mu _v$), human-to-human transmission rate ($\beta$), baseline value of mosquito-to-human transmission rate ($\alpha _h$) and baseline value of human-to-mosquito transmission rate ($\alpha _v$), respectively, in Fig. 8.

The calculations show that the time-average basic reproduction number $[{\mathscr {R}}_0]$ is always less than the basic reproduction ratio ${\mathscr {R}}_0$, suggesting that the time-average basic reproduction number underestimates the disease transmission risk. From this aspect, our results are similar to those of Wang and Zhao (2008). We note that there are some other cases of underestimation and overestimation for the average basic reproduction number can be found in (Bacaër 2007), where an approximate formula of the basic reproduction number was obtained for a class of periodic vector-borne disease models with a small perturbation parameter.

## Discussion

We have developed a compartmental population model to describe the transmission of Zika virus disease in a periodic environment (by including periodic coefficients). We have shown that the global dynamics of the model is determined by the basic reproduction number ${\mathscr {R}}_0$. For ${\mathscr {R}}_0$ less than 1, we have shown the global asymptotic stability of the disease-free periodic solution $E_0$, while the disease persists if ${\mathscr {R}}_0 > 1$. Using our model and taking Ecuador and Colombia as two examples, the fitted curves match the data very well (see Fig.  2). Our numerical simulations suggest that there exists a single positive periodic solution which is globally asymptotically stable for ${\mathscr {R}}_0 > 1$ (see Fig.  3).

The reproduction numbers were calculated as a function of the parameters $\mu _v$, $\alpha _h$, $\alpha _v$ and $\beta$. As is observed, the time-average basic reproduction number $[{\mathscr {R}}_0]$ is always less than the basic reproduction number ${\mathscr {R}}_0$ (see Fig.  8). This implies that the time-average basic reproduction number underrates the risk of disease transmission, while the risk of infection is overestimated by the basic reproduction number.

Although a regular periodic recurrence of Zika has not been observed so far, it is expected that this might be altered by climate change. Our model allows us to estimate what kind of parameter changes might lead to a periodic recurrence of Zika. Using numerical simulations, we found that mosquito birth and death rates are the most significant factors in a possible periodic recurrence of Zika, however, sexual transmission also has a significant effect on the prevalence of the disease.
